# Cross-sectional assessment of anxiety and insomnia of Spanish veterinarians following African swine fever wild boar detection in Spain

**DOI:** 10.3389/fvets.2026.1961344

**Published:** 2026-10-15

**Authors:** Rachel A. Schambow, Paulina Guerra, Marta Martinez Aviles, Irene Iglesias, Antonio Palomo Yagüe, Miguel A. Higuera, Alejandro D. Perez, Andres M. Perez

**Affiliations:** 1 Center for Animal Health and Food Safety, College of Veterinary Medicine, University of Minnesota, St. Paul, MN, United States; 2 Facultad de Ciencias de la Salud, Universidad Argentina de la Empresa (UADE), Buenos Aires, Argentina; 3 Facultad de Ciencias Biomédicas, Universidad Austral, Pilar, Argentina; 4 Centro de Investigacion en Sanidad Animal (CISA-INIA), CSIC, Madrid, Spain; 5 Department of Animal Medicine and Surgery, Faculty of Veterinary Sciences, Complutense University of Madrid, Madrid, Spain; 6 Spanish Pig Producers Association (ANPROGAPOR), Madrid, Spain; 7 Department of Animal Production, University of Veterinary Medicine Alfonso X El Sabio, Madrid, Spain; 8 Doctor Armando Zamudio Hospital, Comandante Luis Piedrabuena, Argentina

**Keywords:** African swine fever, anxiety, insomnia, occupational mental health, Spain, veterinarians

## Abstract

In November 2025, African swine fever (ASF) was detected in wild boar in the Catalonia region of Spain. The primary objective of this cross-sectional survey study was to descriptively explore the mental health impacts of the emergence of ASF in Spain on Spanish practicing veterinarians working with swine and to provide a baseline for future comparison if conditions of the ASF epidemic in Spain significantly change. A questionnaire with the Generalized Anxiety Disorder-7 and Insomnia Severity Index was distributed via convenience sampling to Spanish pig veterinarians from April to May 2026. Working in an ASF control zone, and working in Catalonia, were primary exposures of interest for anxiety and insomnia score. Data were analyzed using univariate analyses. Fifty-three veterinarians responded to the survey. Median anxiety score was 6.00 (interquartile range [IQR]: 2.00–10.0) and median insomnia score was 8.00 (IQR: 4.00–13.0). Some veterinarians reported new feelings of uneasiness or sadness (54.7%), extreme changes in their feelings of happiness and sadness (41.5%), and less confidence in their abilities or their value (41.5%) since the ASF outbreak. Working in an ASF control zone or working in Catalonia were not significantly associated with insomnia or anxiety score (*p* > 0.05). These findings help to describe the current status of anxiety and insomnia amongst Spanish pig veterinarians following the ASF detection in wild boar, and provide a baseline for future comparisons if the ASF epidemic worsens in the country.

## 1 Introduction

Workplace stress is a well-known reality of the veterinary profession. Workplace or occupational stress can be defined as “the harmful physical and emotional responses that occur when the requirements of the job do not match the capabilities, resources, or needs of the worker” (1). Veterinarians are at uniquely high risk for experiencing workplace and occupational-related mental health impacts. Compared to other professions and the general public, mental health in veterinarians tends to be worse. Previous work assessing mental health outcomes mainly amongst practicing veterinarians has identified increased prevalence of depression, anxiety, and stress, which is affected by gender, years of experience, the type of veterinary work or specialty, amongst other factors (2–8). Fewer years of experience and female gender has been associated with worse mental health in multiple studies (2–4). Especially alarming is the observed increased suicide rate among veterinarians compared to other medical professionals and to the general population of many countries (5, 6, 9–11). Burnout has also commonly been reported amongst veterinarians (2, 7).

During an emergency disease response, particularly for transboundary, notifiable diseases with large animal health and socioeconomic impacts, veterinarians are one of many groups at-risk for negative mental health impacts. Veterinarians are typically involved in many frontline activities, such as working with both government officials and clients to manage and maintain farm quarantines or movement restrictions and participating in mass depopulation of animals to prevent disease spread. In particular, mass depopulation, or mass culling, has previously been shown to cause emotional conflict with veterinarians’ professional and ethical obligation to heal animals (12–14). Increased stress, strain, and in some cases long-term effects like post-traumatic stress disorder following emergency disease response have previously been documented during the foot-and-mouth disease outbreaks in the UK in 2001 and in Japan in 2010, during highly pathogenic avian influenza (HPAI) outbreaks in Canada in 2023–2024, and during African swine fever (ASF) outbreaks in the Philippines and Dominican Republic in 2023 and 2024 (12, 15–18). For an occupation that is already under increased risk, the increased stress and strain of emergency disease response could be particularly harmful and lead to development of long-term issues.

In November 2025, ASF, a transboundary and notifiable disease of swine, was detected in the Catalonia region of Spain in wild boar (19). Spain is Europe’s largest producer and exporter of pork, including highly valued Iberian pork, and Catalonia is Spain’s biggest producing region with an estimated 2 million tonnes in pork production and more than 22 million pig heads in 2025 (20, 21). In 2024, Spain exported over 2.76 million tonnes of pork with an estimated value of €8.78 billion (22). Of this, about 26% is exported to non-European Union countries (22). The Spanish government responded with emergency measures aimed at containing and controlling ASF, including zoning and fencing around the outbreaks, testing and removal of wild pig carcasses, general prohibition of movement of domestic pigs and products from the infected zone, and increased biosecurity and surveillance (23, 24). Though domestic pigs have not been affected, the detection in wild boar triggered numerous export bans or trade restrictions based on regionalization (25). As of April 1, 2026, when this study was conducted, there were 42 outbreaks identified in wild boar affecting 241 wild pigs in 10 municipalities of Catalonia, and ASF has not been detected in any domestic pigs in Spain (26).

As transboundary diseases continue to spread necessitating emergency response, the need for mental health preparedness and support for veterinarians who may be impacted by the disease or disease response continues to grow. Preparedness and training prior to participation in these activities could help to reduce potential impacts to veterinarians and other responders; thus, understanding both the mental impacts of outbreaks and baseline measures of the intended population for training are important for ultimately creating context-specific and appropriate preparedness programs. The primary objective of this study was to descriptively explore the anxiety and insomnia of Spanish practicing veterinarians working with swine following the November 2025 detection of ASF in wild boar and to provide a baseline for future comparison if the ASF epidemic in Spain worsens or spreads to domestic pigs. This work will aid in the development of mental health support for Spanish pig veterinarians before, during, and after emergency disease response.

## 2 Methods

This cross-sectional survey study was approved by the University of Minnesota Institutional Review Board (STUDY00020636). The study population was defined as any veterinarians working with swine or the ASF response in Spain. A questionnaire was designed using multiple choice, multiple selection, short answer, and Likert scale questions to capture information on the potential impacts of the ASF emergency on Spanish pig veterinarians (Supplementary File 1, English translation). The Generalized Anxiety Disorder-7 (GAD-7) and Insomnia Severity Index (ISI) were included in the questionnaire to assess signs and symptoms of anxiety and insomnia, respectively (27, 28). Questions previously used on questionnaires to assess the impacts of ASF emergency response on Filipino and Dominican Republic veterinarians were also included (17, 18). Finally, questions were included to understand respondents’ professional experience, their practice area, and demographic characteristics. Already-validated translations for Spanish were used for GAD-7 and ISI items (29, 30), and all other materials were translated into Spanish by members of the research team who were native-Spanish speakers. An online version of the survey was made using Qualtrics. Within the Qualtrics questionnaire, all respondents were given a downloadable, electronic consent form, and consent to participate was given by selecting “Yes” to a mandatory Yes/No question. If “No” were selected, the survey was immediately concluded.

The survey was distributed online from April 1, 2026 through May 30, 2026 to veterinarians working with pigs in Spain. The questionnaire was distributed by email and WhatsApp to the Presidents of the Swine Veterinarians’ Associations, comprised by 5 Regional Associations (Catalonia, Aragon, Galicia, Castile-and-Leon, Murcia), the Iberian Pig Vets Association, the National Pig Vets Association, the National Association of Scientific Pig Farming, and by email to official veterinarians. A reminder message was sent in late April. The questionnaire was also advertised at several in-person meetings held between the President of the Swine Producers’ Association and veterinarians from the affected area. Because recruitment was conducted indirectly through multiple professional associations and overlapping email, WhatsApp and meeting-based channels, the investigators did not have access to a roster of unique eligible recipients. Consequently, the number of individuals who received the invitation and the participation rate could not be determined. Security features in Qualtrics were used to prevent duplicate responses.

Data were downloaded from Qualtrics and imported into R version 4.4.2 for cleaning and analysis. Data were cleaned by removing responses with less than 75% completion. Composite scores were calculated for anxiety and insomnia. The anxiety and insomnia scores were the primary outcomes assessed, while all other responses were exploratory. Scores were only calculated for a given individual if they had completed all items, i.e., those with missing responses to any scored items were excluded. All respondents completed all items for both the GAD-7 and ISI. For anxiety score, each item of the GAD-7 was assigned a score of 0 to 3 (Never = 0, Some days = 1, More than half of days = 2, and Almost every day = 3), then summed across all seven items, giving a score ranging from 0–21 (28). For insomnia score, each item of the ISI was assigned a score as follows: None = 0, Little = 1, Moderate = 2, Severe = 3, Very severe = 4; Very satisfied = 0, Satisfied = 1, Moderately satisfied = 2, Dissatisfied = 3, Very dissatisfied = 4; Not noticeable = 0, A little noticeable = 1, Moderately noticeable = 2, Very noticeable = 3, Too noticeable = 4; Not at all worried = 0, A little worried = 1, Moderately worried = 2, Very worried = 3, Too worried = 4; Does not interfere = 0, Interferes a little = 1, Interferes moderately = 2, Interferes a lot = 3, Interferes too much = 4, giving a score ranging from 0–28 (27). Cronbach’s alpha was used to assess internal consistency of the composite scores (31). Anxiety score was categorized as minimal = 0–4, mild = 5–9, moderate = 10–14, and severe = 15–21 (28). Insomnia score was categorized as no insomnia = 0–7, sub-threshold insomnia = 8–14, moderate insomnia = 15–21, and severe insomnia = 22–28 (27).

Descriptive statistics were performed as counts, histograms, and summary statistics. Univariate analysis was used to assess associations between variables and anxiety score, insomnia score, and symptom presence/absence. Relating to the main objective, two main variables were explored: (1) having worked on any farms in an ASF control area or with producers who have a farm in an ASF control area, and (2) working in the Catalonia region compared to working in the rest of Spain. Age, gender, education, and years of veterinary experience were also explored as potentially associated variables. Because data were not normally distributed and sample size was small, nonparametric Wilcoxon rank-sum test and Kruskal–Wallis tests were used for score outcomes. Pairwise Wilcoxon rank-sum tests with Bonferroni correction were used for post-hoc analysis of significant Kruskal–Wallis associations (*p* < 0.05). Contingency tables and Fisher’s Exact tests were for presence/absence of specific symptoms, and odds ratios (OR) were calculated directly from contingency tables with 95% confidence intervals (CI) calculated using the Wald method.

To descriptively explore potential clusters of respondents, which could ultimately inform development of tailored support and outreach strategies, factor analysis of mixed data (FAMD) and hierarchical cluster analysis on principal components (HCPC) were used (32–34). These were implemented using the FactoMineR and factoextra packages in R (32, 33). Only fully complete responses can be used for FAMD-HCPC, so 45 observations were available for the cluster analysis. The FAMD was conducted using 27 variables (Supplementary File 2). Eigenvalues and a scree plot were examined to explore the total variance explained by each additional principal component dimension. Elbows were identified after 3, 5, and 8 principal dimensions, and HCPC was conducted using each number of dimensions, respectively. HCPC was performed using Euclidian distance, Ward’s agglomerative method, and consolidation using k-means clustering with a maximum of 10 iterations. Three dimensions were chosen for the final HCPC analysis after assessing cluster stability and interpretability (Supplementary File 2). Five and eight dimensions resulted in many small clusters which prevented meaningful interpretation; further details on cluster stability are available through Sankey/Alluvial plots and Adjusted Rand Index Values in Supplementary File 2. Significant describing variables as identified through the HCPC analysis were used to examine and describe each cluster.

## 3 Results

Survey respondent demographics, anxiety score, and insomnia score are described in Table 1. In total, there were 53 responses. Respondents were mainly male (59.2%), aged 46 years and older (71.4%), with more than 15 years of working experience (77.4%), and with highest level of education as “Licenciatura universitaria” or “Grado universitario” (65.3%), equivalent to a 5-year University Degree or 300 European Credit Transfer and Accumulation System (ECTS). “Licenciatura universitaria” is an older denomination for the veterinary university degree in Spain, for which “Grado universitario” is now equivalent. Both are approximately equivalent to a Bachelor of Science degree, while Master universitario and Doctorado are advanced degrees at the Master’s and Doctor’s level, respectively. The majority of respondents were not working in an ASF control zone (79.2%) or were not working in Catalonia (62.3%). Six were working both in a control zone and in Catalonia (11.3%), and 28 were not working in either (52.8%). Six (11.3%) were swine producers in addition to being veterinarians.

**Table 1:** Respondent characteristics and anxiety and insomnia score by strata.

Variable	Variable level	Count (%)	Anxiety score median (IQR)	Insomnia score median (IQR)
Overall (*n* = 53)		6.00 (2.00–10.0)		8.00 (4.00–13.0)
Age (*n* = 49)				
	26–35	9 (18.4)	9.00 (8.00–21.0)	11.0 (6.00–14.0)
	36–45	5 (10.2)	5.00 (1.00–6.00)	10.0 (2.00–12.0)
	46–55	20 (40.8)	6.00 (5.00–10.2)	9.00 (5.75–13.2)
	56 years or more	15 (30.6)	2.00 (0.50–6.00)	4.00 (3.00–12.5)
Gender (*n* = 49)				
	Female	18 (36.7)	7.50 (6.00–11.0)	10.5 (6.00–14.0)
	Male	29 (59.2)	4.00 (1.00–8.00)	6.00 (3.00–12.0)
	Prefer not to respond	2 (4.1)	19.0 (18.0–20.0)	20.0 (19.0–21.0)
Highest education level (*n* = 49)				
	Licenciatura universitaria/Grado universitario (Bachelor-level degree)	32 (65.3)	6.00 (1.00–17.00)	8.50 (4.75–14.0)
	Algunos trabajos de posgrado universitario (Some post-veterinary degree academic work)	4 (8.16)	8.50 (7.50–9.50)	6.50 (3.75–10.5)
	Master universitario (Master’s degree)	8 (16.3)	4.50 (0.75–6.50)	7.00 (1.75–11.2)
	Doctorado (Doctoral degree)	5 (10.2)	7.00 (0.00–7.00)	10.0 (2.00–13.0)
Years of veterinary experience (*n* = 53)				
	1 to 5 years	4 (7.55)	19.5 (15.0–21.0)	8.50 (5.25–13.8)
	6 to 10 years	4 (7.55)	8.50 (6.25–11.5)	12.5 (10.5–14.0)
	11 to 15 years	4 (7.55)	7.50 (5.75–12.0)	11.0 (8.25–16.0)
	More than 15 years	41 (77.4)	6.00 (2.00–8.00)	6.00 (3.00–13.0)
Working in an ASF control zone (*n* = 53)				
	Yes	11 (20.8)	5.00 (2.50–17.5)	9.00 (6.00–16.0)
	No	42 (79.2)	6.00 (2.00–9.00)	7.50 (3.00–12.8)
Working in Catalonia (*n* = 53)				
	Yes	20 (37.7)	5.50 (3.50–12.5)	9.50 (4.00–14.0)
	No	33 (62.3)	6.00 (2.00–9.00)	7.00 (3.00–12.0)

Higher scores indicate a less favorable condition. IQR, Interquartile range.

Cronbach’s alpha was 0.96 for anxiety score and 0.93 for insomnia score. Median anxiety score was 6.00 (interquartile range [IQR] = 2.00–10.0, n = 53, mean = 7.72, standard deviation [SD]: 6.54, range = 0–21, Table 1), and median insomnia score was 8.00, IQR = 4.00–13.0, *n* = 53, mean = 8.98, SD: 6.32, range = 0–28, Table 1). By anxiety category, 18 (34.0%) were categorized as minimal anxiety, 21 (39.6%) as mild, 3 (5.67%) as moderate, and 11 (20.8%) as severe. By insomnia category, 26 (49.1%) were categorized as no insomnia, 18 (34.0%) as sub-threshold, 7 (13.2%) as moderate, and 2 (3.77%) as severe. Individual counts and frequencies by GAD-7 and ISI items are available in Tables 2, 3. Anxiety and insomnia score were not significantly different for those working in an ASF control zone (anxiety: *n* = 53, *W* = 227.5 and *p* = 0.95; insomnia: *n* = 53, *W* = 169.5 and *p* = 0.18) or working in Catalonia (anxiety: *n* = 53 *W* = 329.5 and *p* = 1; insomnia: *n* = 53, *W* = 280 and *p* = 0.36). The Kruskal–Wallis tests indicated a significant difference in anxiety scores across age groups (*χ*^2^ = 11.2, *p* = 0.01, *n* = 49) and gender groups (*χ*^2^ = 11.4, *p* = 0.003, *n* = 49), and in insomnia scores across gender groups (*χ*^2^ = 8.09, *p* = 0.02, *n* = 49). Follow-up pairwise Wilcoxon rank-sum tests for age category was not significant following Bonferroni correction for anxiety score (*p* > =0.07), and was also not significant for insomnia score and gender following Bonferroni correction (*p* > =0.1). Females had significantly higher anxiety scores compared to males after Bonferroni correction (Table 1, adjusted *p* = 0.01).

**Table 2:** Count and frequencies of responses to general anxiety disorder-7 (GAD-7) questions (*n* = 53).

GAD-7 item	Never	Several days	More than half	Almost every day
Feeling nervous, anxious, or on edge	18 (34.0%)	19 (35.8%)	5 (9.43%)	11 (20.8%)
Not being able to stop worrying or not being able to control worry	16 (30.2%)	23 (43.4%)	5 (9.43%)	9 (17.0%)
Worrying too much about different things	10 (18.9%)	26 (49.1%)	8 (15.1%)	9 (17.0%)
Difficulty relaxing	13 (24.5%)	24 (45.3%)	8 (15.1%)	8 (15.1%)
Being so restless that it is hard to sit still	24 (45.3%)	18 (34.0%)	4 (7.55%)	7 (13.2%)
Getting upset or irritated easily	15 (28.3%)	22 (41.5%)	6 (11.3%)	10 (18.9%)
Feeling scared like something terrible might happen	26 (49.1%)	18 (34.0%)	2 (3.77%)	7 (13.2%)

**Table 3:** Count and frequencies of responses to insomnia severity index (ISI) questions (*n* = 53).

ISI item	Response level number of respondents (%)				
Difficulty sleeping	*None*	*Little*	*Moderate*	*Severe*	*Very severe*
	14 (26.4%)	25 (47.2%)	11 (20.8%)	2 (3.77%)	1 (1.89%)
Difficulty staying asleep	18 (34.0%)	15 (28.3%)	13 (24.5%)	5 (9.43%)	2 (3.77%)
I wake up very early	10 (18.9%)	6 (11.3%)	23 (43.4%)	12 (22.6%)	2 (3.77%)
How satisfied/dissatisfied are you with your current sleep pattern?	*Very dissatisfied*	*Dissatisfied*	*Moderately satisfied*	*Satisfied*	*Very satisfied*
	2 (3.77%)	11 (20.8%)	19 (35.8%)	17 (32.1%)	4 (7.55%)
How noticeable to others do you think your sleep problem is in terms of deteriorating your quality of life?	*Not noticeable*	*A little noticeable*	*Moderately noticeable*	*Very noticeable*	*Too noticeable*
	25 (47.2%)	10 (18.9%)	10 (18.9%)	7 (13.2%)	1 (1.89%)
How worried / distressed are you about your current sleep problem?	*Not at all worried*	*A little worried*	*Moderately worried*	*Very worried*	*Too worried*
	27 (50.9%)	13 (24.5%)	9 (17.0%)	3 (5.67%)	1 (1.89%)
To what extent do you feel your sleep problem interferes with your daily functioning (e.g., daytime fatigue, mood, ability to function at work/daily tasks, concentration, memory, mood, etc.) currently?	*Does not interfere*	*Interferes a little*	*Interferes moderately*	*Interferes a lot*	*Interferes too much*
	19 (35.8%)	16 (30.2%)	10 (18.9%)	4 (7.55%)	4 (7.55%)

The most commonly reported symptoms following the ASF outbreaks were new feelings of uneasiness or sadness (Table 4, 54.7%), extreme changes in their feelings of happiness and sadness (41.5%), and less confidence in their abilities or their value (41.5%). Age category was significantly associated with reduced energy (*p* = 0.04, *n* = 49), not feeling positive for the future (*p* = 0.04, *n* = 49), feelings of anger or frustration (*p* = 0.03, *n* = 49), and less confidence (*p* = 0.005, *n* = 49). Specifically, compared to the reference category of respondents 56 or older, those 26–35 years old were more likely to report reduced energy (OR: 17.5, 95% CI: 1.56–196, *n* = 49) and less confidence (OR: 32.0, 95% CI: 2.81–365, *n* = 49) and those 46–55 years old were more likely to report not feeling positive for the future (OR: 5.96, 95% CI: 1.33–26.7, *n* = 49) and feelings of anger or frustration (4.89, 95% CI: 1.05–22.8, *n* = 49). Additionally, respondents with 6–10 years of veterinary experience were significantly more likely to report reduced energy compared to those with more than 15 years of experience (*p* = 0.03; OR: 14.6, 95% CI: 1.32–161, *n* = 49). All respondents that reported 6–10 years of experience were also 26–35 years of age.

**Table 4:** Count and frequencies of reported symptoms following the African swine fever detection in Spain.

Question (number of respondents)	Number of yes responses (percent)				
Do you use medication to sleep or fall asleep? (*n* = 53)	5 (9.43%)				
Do you use medication to feel calmer? (*n* = 52)	5 (9.61%)				
Do you feel positive about the future since the ASF outbreak? (*n* = 52)	25 (48.1%)				
Have you experienced reduced energy since the ASF outbreak? (*n* = 53)	13 (24.5%)				
Have you experienced reduction in the way you enjoy your life since the ASF outbreak? (*n* = 52)	20 (38.5%)				
Have you experienced new feelings of anger or frustration since the ASF outbreak? (*n* = 53)	21 (39.6%)				
Have you experienced new feelings of uneasiness or sadness since the ASF outbreak? (*n* = 53)	29 (54.7%)				
Do you have trouble concentrating on tasks since the ASF outbreak? (*n* = 53)	18 (34.0%)				
Have you experienced memory problems since the ASF outbreak? (*n* = 53)	17 (32.1%)				
Have you experienced extreme changes in your feelings of happiness and sadness since the ASF outbreak? (*n* = 53)	22 (41.5%)				
Do you have less confidence in your abilities or their value since the ASF outbreak? (*n* = 53)	22 (41.5%)				
Have you found it difficult to maintain your social activities, such as visiting friends or family, since the ASF outbreak? (*n* = 52)	12 (22.6%)				
Were you already seeing a mental health professional before the ASF outbreaks? (*n* = 53)	5 (9.43%)				
Have you started or increased your visits to a mental health professional since the outbreak? (*n* = 52)	0 (0%)				
Have you experienced any negative behaviors from your neighbors or social circle since the outbreak? (*n* = 53)	4 (7.55%)				
Have you experienced any changes in your physical exercise? (*n* = 49)	*Less*			*Same*	*More*
	7 (14.3%)			38 (77.6%)	4 (8.16%)
Has the number of farms you visit, on average, per day changed since before the outbreaks? (*n* = 49)	8 (16.3%)			40 (81.6%)	1 (2.04%)
How much has your job satisfaction changed? (*n* = 49)	*Much worse*	*A little worse*	*Same*	*A little better*	*Much better*
	5 (10.2%)	15 (30.6%)	28 (57.1%)	0 (0%)	1 (2.04%)
How has your behavior changed in society and the community? (*n* = 49)	*Wasn’t involved*	*Less*	*No change*	*More*	
	0 (0%)	6 (12.2%)	38 (77.6%)	5 (10.2%)	

These questions do not constitute a validated diagnostic scale.

The FAMD and HCPC analyses identified 4 exploratory, descriptive clusters (*n* = 45). Overall mean scores and proportions are of the 45 responses used for cluster analysis, and comparison is provided to describe each cluster’s composition. Cluster composition is available in Supplementary File 3.

Cluster 1 had 20 members and was characterized by significantly lower insomnia (4.45 vs. 8.78) and anxiety scores (2.7 vs. 7.36) than the overall cluster analysis mean, and significantly lower symptoms of anger or frustration (0% vs. 37.8%), reduced enjoyment of life (5.0% vs. 40%), extreme changes in feelings (10.0% vs. 44.4%), unease or sadness (25% vs. 57.8%), trouble concentrating (5% vs. 33.3%), reduced energy (0% vs. 24.4%), reduced confidence (15% vs. 44.4%), difficulty doing social activities (5% vs. 22.2%), and poor memory (15% vs. 33.3%). A significantly higher proportion also reported feeling positive for the future (75% vs. 53.3%). Job satisfaction was mostly similar to prior to the ASF outbreaks (85% vs. 60%), with none reporting worse job satisfaction. This cluster was significantly comprised of males (90.0% vs. 62.2%) and those 56 + years of age (60% vs. 33.3%). All had 15 or more years of experience as veterinarians, and all that had doctoral degrees (*n* = 5) were in this cluster.

Cluster 2 had 6 members, and insomnia and anxiety scores did not differ significantly from mean values. All reported similar job satisfaction to pre-ASF outbreaks, and all reported no feelings of anger or frustration. Fifty percent of the cluster was veterinarians with 11–15 years of experience and 67% were 26–35 years old. All veterinarians in this cluster had less than 15 years of experience.

Cluster 3 had 14 members with significantly higher insomnia (13.4 vs. 8.78) and anxiety scores (11.5 vs. 7.36) than the mean value. They also reported higher frequencies of anger or frustration (92.9% vs. 37.8%), unease or sadness (100% vs. 57.8%), extreme changes in feelings (85.7% vs. 44.4%), reduced enjoyment in life (78.6% vs. 40%), trouble concentrating (64.3% vs. 33.3%), poor memory (57.1% vs. 33.3%), and difficulty doing social activities (42.9% vs. 22.2%). They also had significantly higher proportion of those reporting worse job satisfaction (28.6% reporting “much worse” vs. 11.1%) and lower proportion of feeling positive for the future (28.6% vs. 53.3%). The cluster was mainly veterinarians aged 46–55 (78.6%), with no vets aged 26–35 in this cluster. All had more than 15 years of experience.

Cluster 4 had 5 members with a significantly higher anxiety score compared to the mean (16 vs. 7.36), but insomnia score was not significantly different. The cluster also had increased frequency of reduced enjoyment of life (100% vs. 40%), reduced energy (80% vs. 24.4%), extreme changes in feelings (100% vs. 44.4%), and trouble concentrating (80% vs. 33.3%). This cluster was entirely comprised of veterinarians aged 26–45, 26–35 (80%), and had more veterinarians that reported having previously had visits with a mental health professional (60% vs. 11.1%). This cluster was entirely composed of women and those who chose not to specify their gender.

## 4 Discussion

This brief report summarizes anxiety and insomnia scores from veterinarians working in the Spanish pig industry after the outbreak of ASF in wild boar. Self-reported anxiety via the GAD-7 in this sample of veterinarians varied, though approximately 26% were in moderate or severe categories; by comparison, 17% were in moderate to severe categories of self-reported insomnia. Some work has been done to characterize mental health in Spain during the COVID-19 pandemic, which provides some comparison for these results. A study of anxiety amongst healthcare workers in Spain found a high prevalence of anxiety associated with years of experience, workload, and other factors, and which showed a clear decrease from a mean of 10.82 to 7.61 across two years measured by the GAD-7 (35). In another study of this population from the same authors where insomnia was evaluated through the ISI, they found a similar decrease in insomnia score from a mean of 11.72 to 9.76 across the two year period (36). Other studies conducted during the COVID-19 pandemic found a mean GAD-7 score of 7.72 (SD: 5.17) amongst a sample of the Spanish general population, and of pregnant or postpartum women in Spain, 33.3% of the respondents had GAD-7 scores of 10 or greater (classified as moderate or severe) (37, 38). It is difficult to compare the levels reported here to what may be baseline levels amongst the Spanish general population during non-emergency conditions; however, these results will be beneficial as a baseline for future comparison of anxiety and insomnia in this specific sub-population of Spanish veterinarians if conditions relating to the ASF outbreak in Spain worsened.

Proportionally, the highest reported symptoms related to the ASF outbreaks were related to unease and low confidence (Table 4), which could potentially be alleviated through additional training and support for veterinarians to understand their role in ASF control measures. Regarding the main objective of the study, there were no statistically significant differences in mental health outcomes for veterinarians working within an ASF control zone compared to those not within a control zone, or for those working in Catalonia compared to those working in the rest of Spain. This could be due to numerous reasons, such as the small sample size and small number of individuals working in the affected areas, which is reflective of the small number of pig premises within the affected area, and ASF only being detected in wild boar and not domestic pigs (23). It is possible that if the ASF outbreaks expand geographically or into domestic pigs, more significant mental health impacts could be observed. The present findings should be interpreted in the context of a phase of heightened alert and preparedness while ASF is only present in wild boar, rather than reflecting the direct psychological impact of managing ASF outbreaks on commercial pig farms. It is also possible that these veterinarians may be receiving adequate mental health support, and may be more protected from negative outcomes.

Age appears to be an important variable both in univariate analysis and in cluster analysis, with lower age being associated with more negative mental health outcomes. This is likely also correlated with increased years of experience. Other studies have also found that older veterinarians and those with more years of experience report better mental health levels, as they tend to have greater preparedness to manage stress and engage in self-care activities (2–4). In some cases, it may also be due to lower mental health awareness and therefore less reporting of symptoms (39). It is interesting, however, that this pattern is not observed in Cluster 3, which had a higher proportion of veterinarians 46–55 years old, but still showed worse levels of mental health.

Female gender was significantly associated with a higher anxiety score in univariate analysis. Gender has also been associated with poorer mental health outcomes both in veterinarians (3, 4), including female veterinarians in Spain (40), and in the general population (41, 42). Women report higher levels of stress, more negative work experiences, and greater help-seeking behavior (3). Globally, veterinary medicine has been transitioning from a male-dominated profession to a female-dominated profession, yet despite this, women still report discrimination, unequal pay, and other negative workplace factors that may affect their mental wellbeing (43–45).

There were important limitations to this study. Overall, sample sizes were low resulting in decreased power to show significant differences. It is also difficult to extrapolate the actual intensity of involvement in the response based on geographic location or working in a control zone; future studies that capture direct participation, specific tasks, duration and frequency of involvement, and other information will help to better measure the exposure level to the outbreak response. Qualitative research can help to explore the underlying stressors that may be driving the trends observed here, such as the decreased mental health amongst women, younger veterinarians, and those aged 46–55. The convenience sampling and recruitment strategy may have introduced selection and non-response bias. Because invitations were disseminated through multiple overlapping professional networks and the number and characteristics of unique recipients were unknown, the respondents cannot be assumed to be representative of all veterinarians working with swine in Spain, and a participation rate could not be calculated.

In conclusion, this study provides an initial baseline and preliminary descriptive information for understanding anxiety and insomnia amongst Spanish swine veterinarians during the ongoing ASF outbreak in wild boar. Educational efforts and training exercises may help reduce feelings of unease or low confidence in responding to the ASF situation, which were reported here. These findings underscore the need for targeted mental health support strategies, especially as global impact of transboundary animal diseases continues to evolve, highlighting the necessity of proactive, tailored preparedness measures, and interventions to mitigate the risk of long-term mental health challenges for veterinary practitioners.

## Data Availability

The datasets presented in this article are not readily available because the study protocol that was submitted and approved by the University of Minnesota Institutional Review Board (STUDY00020636) specified that “Only summary data (such as distributions or frequencies of responses) will be shared.” Due to this, we are unable to share individual responses without violating the approved protocol. All summary data for the questionnaire responses have been provided in Tables 1–4. Requests to access the datasets should be directed to University of Minnesota IRB, irb@umn.edu.
