# Supplementary material for: Cross-sectional assessment of anxiety and insomnia of Spanish veterinarians following African swine fever wild boar detection in Spain
Source: Front Vet Sci. 2026 Oct 15;13:1961344. doi: 10.3389/fvets.2026.1961344 (PMC13717630; doi:10.3389/fvets.2026.1961344)
Supplement: Supplementary file 1 [file Data_Sheet_1.pdf]

Supplementary File 1. English translation of survey

Start of Block: Intro_and_Consent

Impacts of African swine fever in Spain You are asked to participate in a research study. This survey is carried out by the Center for Animal Health and Food Safety at the University of Minnesota in collaboration with the National Association of Pig Producers (ANPROGAPOR) and the Center for Research in Animal Health (CISA) of the Spanish National Research Council. The aim of the survey is to better understand the impact of recent cases of African swine fever (ASF) on veterinarians in Spain. Their response is essential to design programs and policies that address the needs of farmers and veterinarians during emergencies such as these. Thank you for your time and valuable feedback.
To participate in this survey, you must work in Spain and be over 18 years of age and be a veterinarian working with production/farm animals

Page Break

You must review and agree to the consent note below before proceeding with the survey. The survey is confidential and completely voluntary. If you do not accept the consent form and do not wish to take the survey, you can exit the survey at this time or answer "No" to the question below

---

Have you read the consent letter and agree to take the survey? Clicking "Yes" will take you to the first question. Clicking "No" will end the survey.

- ○ Yes
- ○ No

Skip To: End of Survey If You have read the consent letter and agree to take the survey? Clicking "Yes" will take you to the ... = No

First, we would like to learn more about your veterinary practice, to help us better understand the veterinarians who answer this questionnaire.

Are you a pig farmer or veterinarian?

- ○ Pig Producer
- ○ Veterinarian
- ○ Both pig producer and veterinarian

How many years have you been practicing veterinary medicine?

- ◯ Less than 1 year
- ◯ 1 to 5 years
- ◯ 6 to 10 years
- ◯ 11 to 15 years old
- ◯ More than 15 years

What species do you work with? Check all that apply.

- ☐ Pork
- ☐ Poultry (chickens, turkeys, or others)
- ☐ Sheep or goats
- ☐ Cattle (beef)
- ☐ Cattle (dairy)
- ☐ Horses
- ☐ Another ____

In which autonomous communities do you work or work most frequently? (Select up to three)

*

- ☐ Andalusia
- ☐ Aragon
- ☐ Canary Islands
- ☐ Cantabria

- [ ] Castilla y León
- [ ] Castilla-La Mancha
- [ ] Catalonia
- [ ] Community of Madrid
- [ ] Autonomous Community of Navarre
- [ ] Valencian Community
- [ ] Extremadura
- [ ] Galicia
- [ ] Balearic Islands
- [ ] La Rioja
- [ ] Basque Country
- [ ] Principality of Asturias

- [ ] Region of Murcia

In which provinces do you work most frequently? (Select up to three)

- [ ] Álava
- [ ] Albacete
- [ ] Alicante
- [ ] Almeria
- [ ] Asturias
- [ ] Avila
- [ ] Badajoz
- [ ] Barcelona
- [ ] Burgos
- [ ] Cáceres
- [ ] Cadiz
- [ ] Cantabria
- [ ] Castellón
- [ ] Ciudad Real
- [ ] Cordoba
- [ ] La Coruña
- [ ] Cuenca
- [ ] Girona
- [ ] Granada
- [ ] Guadalajara

- [ ] Guipúzcoa
- [ ] Huelva
- [ ] Huesca
- [ ] Balearic Islands
- [ ] Jaén
- [ ] León
- [ ] Lleida
- [ ] Lugo
- [ ] Madrid
- [ ] Malaga
- [ ] Murcia
- [ ] Navarre
- [ ] Orense
- [ ] Palencia
- [ ] Las Palmas
- [ ] Pontevedra
- [ ] La Rioja
- [ ] Salamanca
- [ ] Segovia
- [ ] Seville

- [ ] Soria
- [ ] Tarragona
- [ ] Santa Cruz de Tenerife
- [ ] Teruel
- [ ] Toledo
- [ ] Valencia
- [ ] Valladolid
- [ ] Vizcaya
- [ ] Zamora
- [ ] Zaragoza

Have you worked on any farms in an African swine fever control area or with producers who have a farm in an African swine fever control area?

○ Yes

○ No

In the next few sections, we'll ask questions about your well-being. Please, as you answer them, consider how you have felt over the past two weeks.

Over the past 14 days, how often have you felt discomfort from the following issues?

	Never	Several days	More than half of the days	Almost every day
Feeling nervous, anxious, or on edge	○	○	○	○
Not being able to stop worrying or not being able to control worry	○	○	○	○
Worrying too much about different things	○	○	○	○
Difficulty relaxing	○	○	○	○
Being so restless that it is hard to sit still	○	○	○	○
Getting upset or irritated easily	○	○	○	○
Feeling scared like something terrible might happen	○	○	○	○

Page Break

Do you use medication to sleep or fall asleep?

- ◯ Yes
- ◯ No

Display this question:

If you use medication to sleep or fall asleep? = Yes

What medication do you use to fall asleep or fall asleep?

____

Page Break

Do you use medication to feel calmer?

○ Yes

○ No

Display this question:

Do you use medication to feel calmer? = Yes

What medication do you use to feel calmer?

____

Page Break

Rate the CURRENT SEVERITY (i.e., LAST 2 WEEKS) of your insomnia problem(s).

	None	Little	Moderate	Severe	Very severe
Difficulty sleeping	○	○	○	○	○
Difficulty staying asleep	○	○	○	○	○
I wake up very early	○	○	○	○	○

Page Break

How satisfied/dissatisfied are you with your current sleep pattern?

- ◯ Very satisfied
- ◯ Satisfied
- ◯ Moderately satisfied
- ◯ Dissatisfied
- ◯ Very dissatisfied

How REMARKABLE to others do you think your sleep problem is in terms of deteriorating your quality of life?

- ◯ Not remarkable
- ◯ A little noticeable
- ◯ Moderately noticeable
- ◯ Very remarkable
- ◯ Too noticeable

How WORRIED/DISTRESSED are you about your current sleep problem?

- ◯ Nothing worried
- ◯ A little worried
- ◯ Moderately concerned
- ◯ Very concerned
- ◯ Too worried

To what extent do you feel your sleep problem INTERFERES with your daily functioning (e.g., daytime fatigue, mood, ability to function at work/daily tasks, concentration, memory, mood, etc.) CURRENTLY?

- ○ Does not interfere
- ○ Interferes a little
- ○ Interferes moderately
- ○ Interferes a lot
- ○ Interferes too much

End of Block: Sleep

Start of Block: ASF_Questionnaire

For the following questions, since cases of African swine fever (ASF) were reported in wild boar in Catalonia in November 2025:

Have you felt a reduction in your energy?

- ○ Yes
- ○ No

Have you felt a reduction in the way you enjoy life?

- ○ Yes
- ○ No

Do you feel positive about your future?

- ◯ Yes
- ◯ No

Have you felt new feelings of uneasiness or sadness?

- ◯ Yes
- ◯ No

Have you had trouble concentrating or performing your tasks?

- ◯ Yes
- ◯ No

Have you had memory problems?

- ◯ Yes
- ◯ No

Have you experienced new feelings of anger or frustration?

- ◯ Yes
- ◯ No

Have you experienced extreme changes in your feelings of happiness or sadness?

- ◯ Yes
- ◯ No

Have you felt less confident in your abilities or their value?

- ◯ Yes
- ◯ No

Have you found it difficult to maintain your social activities, such as visiting friends or family?

- ◯ Yes
- ◯ No

Were you already seeing a mental health professional before ASF outbreaks?

- ◯ Yes
- ◯ No

Have you started or increased your visits to a mental health professional?

- ◯ Yes
- ◯ No

Have you experienced any negative attitudes or behaviors from your neighbors or social circle?

- Yes
- No

Have you experienced any changes in your physical exercise?

- Less
- More or less the same
- More

How many farms do you visit on average per day?

____

Has the number of farms you visit, on average, per day changed since before the outbreaks?

- Visit more farms
- More or less the same
- Visit fewer farms

How much did their job satisfaction change?

- Much worse
- A little worse
- More or less the same
- A little better
- Much better

How has their behavior changed in society and the community?

- More participation
- No changes
- Less participation
- I was never involved in my community.

Finally, we would like to know a little more about you to better understand the people who completed this questionnaire. Remember that all your answers are confidential.

How old are you?

- ◯ 18 to 25
- ◯ 26 to 35
- ◯ 36 to 45
- ◯ 46 to 55
- ◯ 56 years or older

Which option best describes your gender?

- ◯ Male
- ◯ Female
- ◯ Non-binary/third gender
- ◯ I prefer to describe myself ________________
- ◯ I prefer not to answer

What is the highest level of formal education you have completed?

- He did not finish Compulsory Secondary School (ESO)
- Compulsory Secondary School
- Baccalaureate
- Intermediate Vocational Training
- Higher Level Vocational Training
- Incomplete university studies
- University degree
- Master's degree
- University Bachelor's Degree
- Some university postgraduate work
- Doctorate
- I prefer not to answer
