# Supplementary material for: Cross-sectional assessment of anxiety and insomnia of Spanish veterinarians following African swine fever wild boar detection in Spain
Source: Front Vet Sci. 2026 Oct 15;13:1961344. doi: 10.3389/fvets.2026.1961344 (PMC13717630; doi:10.3389/fvets.2026.1961344)

Supplementary File 2: Sensitivity Analysis for FAMD and HCPC

Variables used in the analysis:

- Total anxiety score
- Total insomnia score
- Occupation (veterinarian or veterinarian/farmer)
- Years of experience as a veterinarian
- Gender
- Age
- Highest education level
- Working in a ASF control zone or not
- Working in Catalonia
- Use medications to sleep or fall asleep
- Use medications to feel calmer
- Reduced energy
- Reduced enjoyment of life
- Feel positive for the future
- New feelings of uneasiness or sadness
- Trouble concentrating or performing their task
- Memory problems
- Less confident in your abilities or their value?
- New feelings of anger or frustration
- Extreme changes in feelings of happiness or sadness
- Difficulty maintaining social activities
- Already seeing a mental health professional before ASF outbreak
- Experienced negative attitudes or behaviors from neighbors or social circle
- Experience any change in physical exercise
- How the number of farms visited on average per day has changed
- Job satisfaction
- How behavior in community has changed

Scree plot of FAMD analysis. X-axis is the number of dimensions, and y-axis is the percentage of explained variance for each dimension.

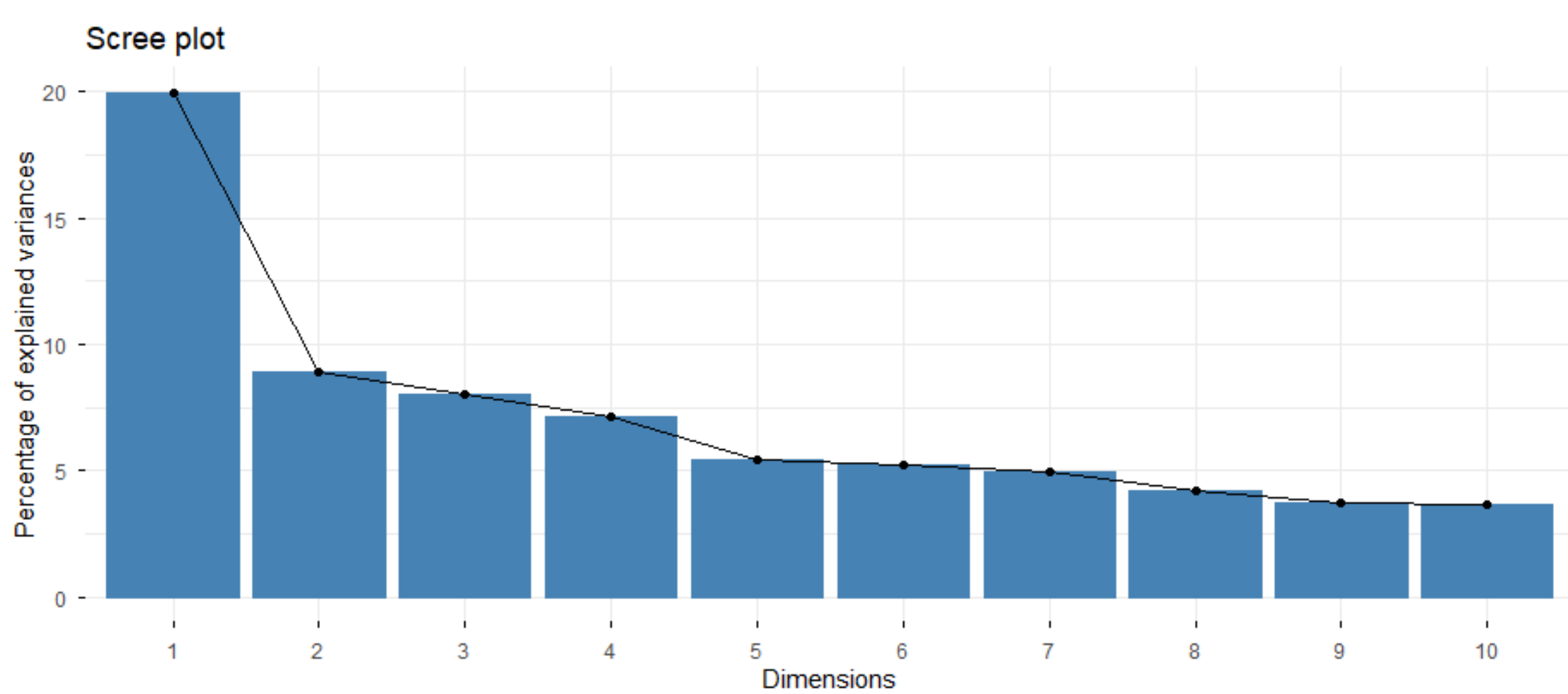

Number of clusters and cluster sizes for HCPC of 3, 5, and 8 principal component dimensions.

Cluster Number	Number of Responses in Cluster		
	3 Dimensions	5 Dimensions	8 Dimensions
1	20	15	14
2	6	5	5
3	14	4	4
4	5	5	5
5	Not present	10	5
6	Not present	3	1
7	Not present	2	6
8	Not present	1	1
9	Not present	Not present	1
10	Not present	Not present	2
11	Not present	Not present	1

Cluster stability: Adjusted Rand Index Values (Values range from 0 to 1, completely unstable to perfectly stable)

Comparison	Value
3 dimensions vs. 5 dimensions	0.509
3 dimensions vs. 8 dimensions	0.481
5 dimensions vs. 8 dimensions	0.782

Sankey/Alluvial plots of cluster stability across 3 to 5, 5 to 8, and 3 to 8 principle component dimensions. Plots show how the cluster assignment of individual responses changes across different models. Cluster numbers are depicted in the vertical stacked white boxes. Colored bars indicate the movement of individuals or groups between cluster assignments in different models.

A) 3 dimensions (left) compared to 5 dimensions (right)

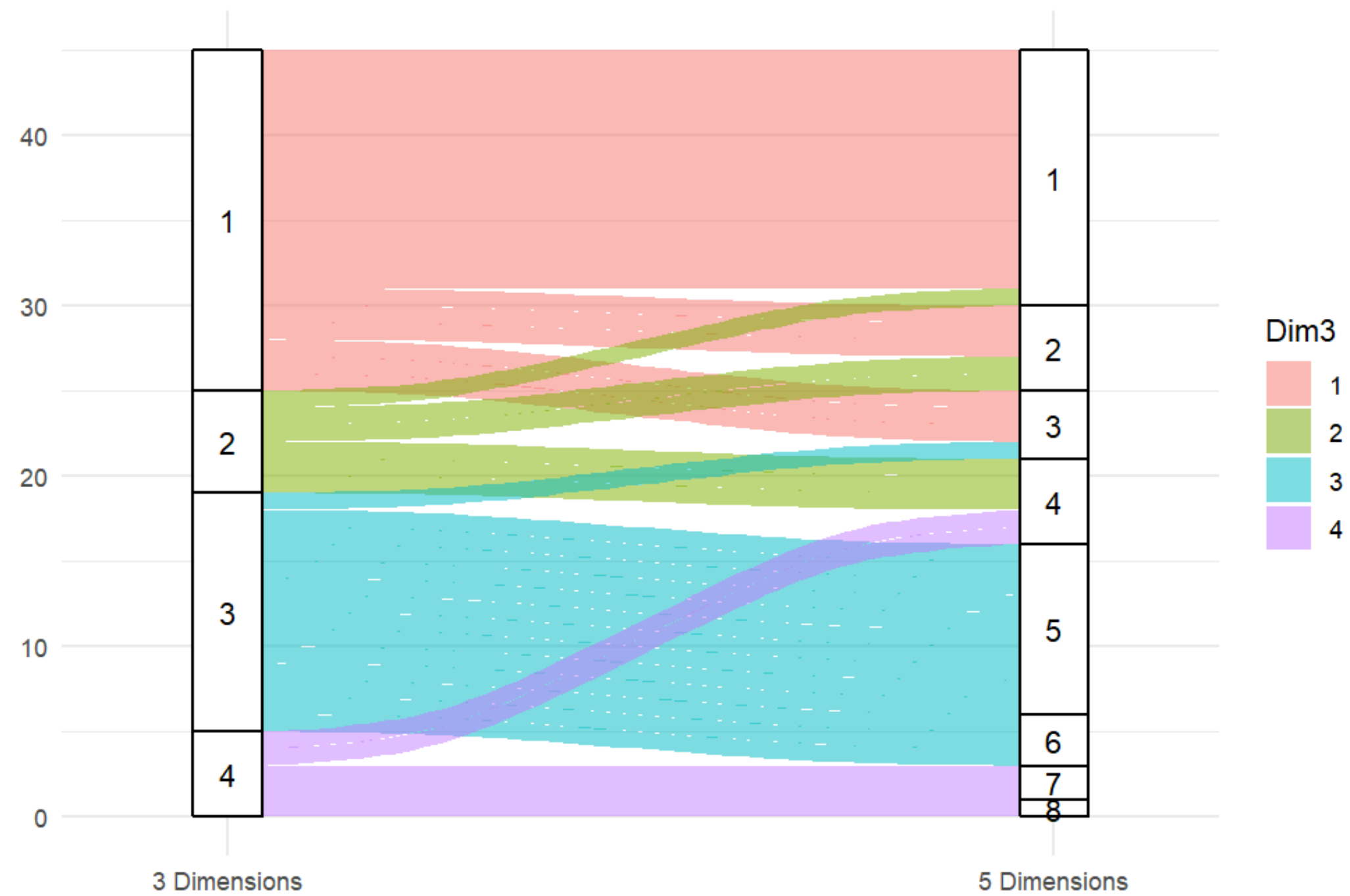

B) 5 dimensions (left) compared to 8 dimensions (right)

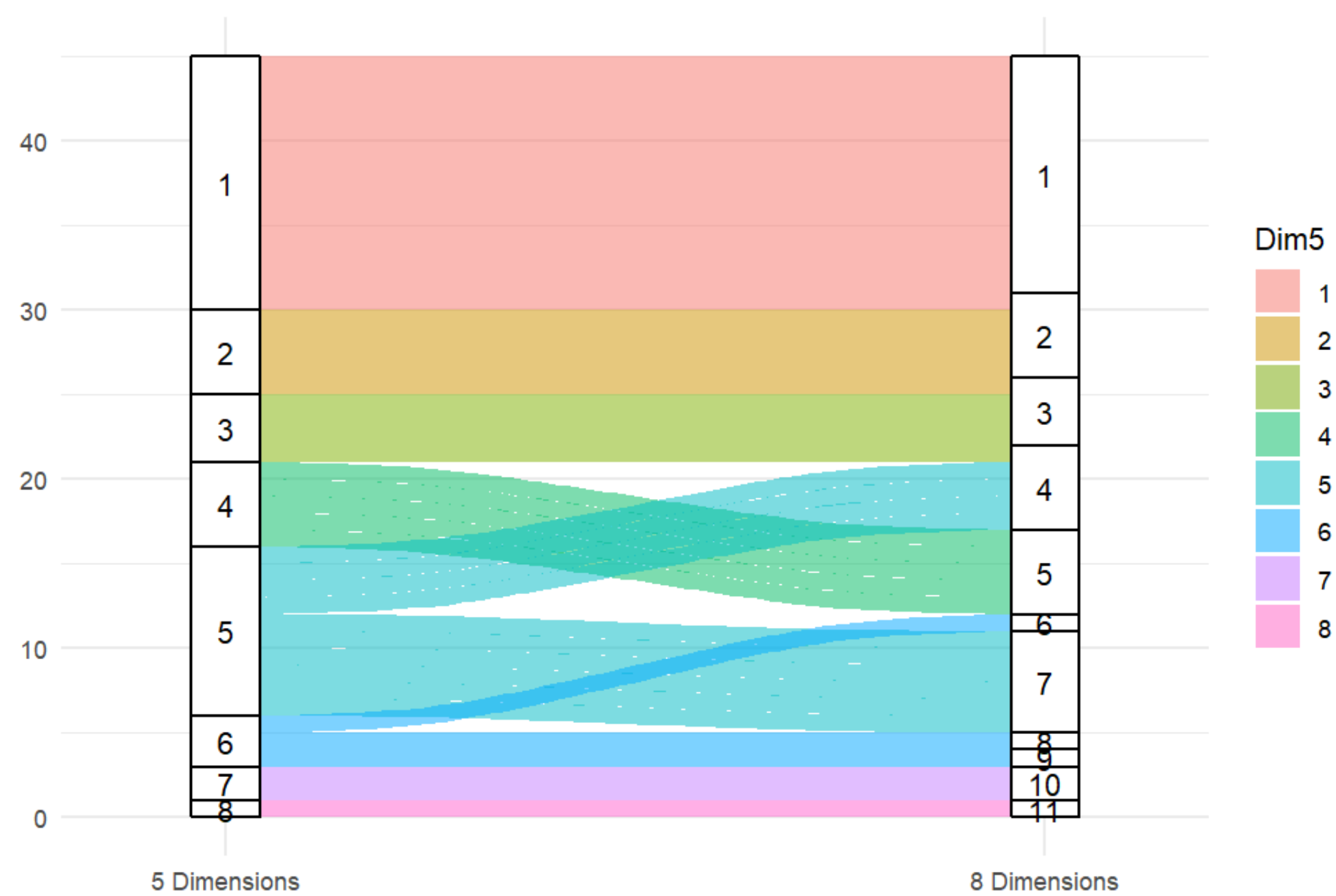

C) 3 dimensions (left) compared to 8 dimensions (right)

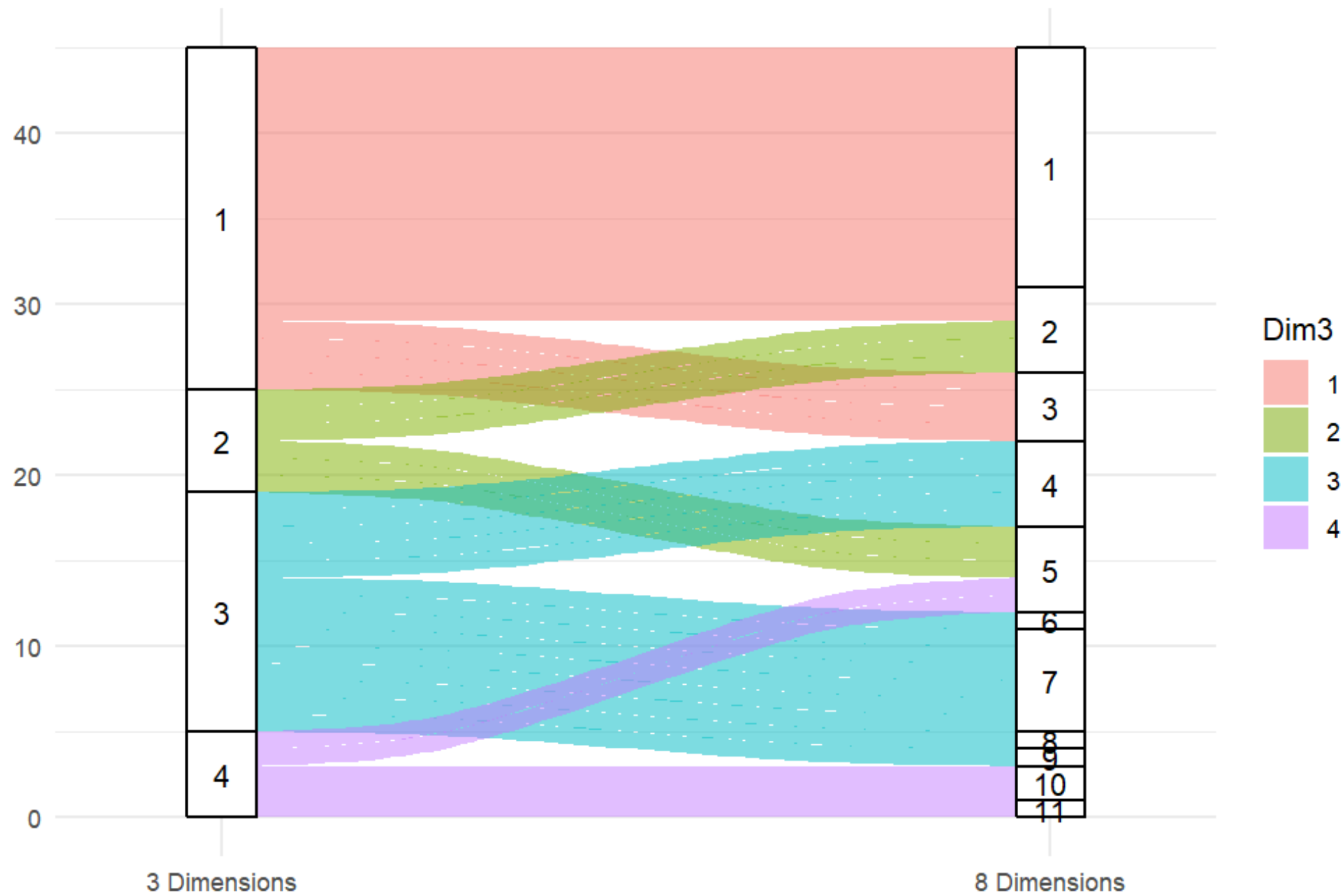

Supplement: Supplementary file 2 [file Data_Sheet_2.pdf]
